# A neuropsychological assessment, using computerized battery tests (CANTAB), in children with benign rolandic epilepsy before AED therapy

**Published:** 2012-03-05

**Authors:** MA Vinţan, S Palade, A Cristea, I Benga, DF Muresanu

**Affiliations:** * “Iuliu Haţieganu” University of Medicine and Pharmacy, Department of Neurosciences, Cluj-Napoca, Romania; ** Children’s Hospital, Division of Pediatric Neurology, Cluj-Napoca, Romania

**Keywords:** benign, rolandic, children, epilepsy, CANTAB

## Abstract

**Rationale: **Benign rolandic epilepsy (BRE) is a form of partial idiopathic epilepsy according to the International League Against Epilepsy (ILAE) syndromes classification (1989). Recent studies have identified cases of BRE that do not meet the initial definition of ‘benign’; these included reports of cases with specific cognitive deficits. It is still a matter of debate, whether these deficits are due to epilepsy per se, to treatment or other associated factors.

**Objectives: **The aim of this study was to evaluate if BRE children have cognitive deficits at the onset of their seizures, prior to their participation in any anti-epileptic drug therapy (AED).

**Methods and Results: **We performed a neuropsychological assessment of 18 BRE children compared with a corresponding age-matched control group. We used the Cambridge Neuropsychological Test Automated Battery (CANTAB). Subjects were at their first neurological evaluation, before any AED therapy. We assessed: visual memory, induction and executive functions. In our group, the BRE children performed comparably with the control children for the induction and executive functions. Substantial differences were identified for the visual memory subtests: PRM percent correct (t = -2.58, p = 0.01) and SRM percent correct (t = -2.73, p = 0.01). Age of seizure onset had a negative impact on the visual memory subtest performances (PRM mean correct latency). We found significant correlations between the different CANTAB subtests results and characteristics of the centrotemporal spikes (CTS).

**Discussion: **Our results are consistent with the findings of other similar studies. This form of epilepsy is associated with subtle neuropsychological deficits, present at seizure onset. Neuropsychological deficits identified, suggest a more diffuse brain involvement in the epileptiform process.

**Abbreviations: **AED – AntiEpileptic Drug; BECTS - benign childhood epilepsy with centrotemporal spikes; BRE – Benign Rolandic Epilepsy; CANTAB - the Cambridge Neuropsychological Test Automated Battery; CTS – centrotemporal spikes; DMS – Delayed Matching Sample; EEG – electroencephalogram; ILAE – International league Against Epilepsy; MOT – Motor screening Test; PAL - Paired Associates Learning; PRM - Pattern Recognition Memory; SPSS - Statistical Package for the Social Sciences; SRM - Spatial Recognition Memory; SSP - Spatial Span, SWM - Spatial Working Memory

## Introduction

Benign rolandic epilepsy (BRE), first described in 1597 by Martinus Rulandus, is a form of partial idiopathic epilepsy, according to the 1989 ILAE Commission of Classification and Terminology classification [**8**]. However, the clinical features and electroencephalographic characteristics have only been recognized in the last 40 years. BRE was initially named *benign childhood epilepsy with centrotemporal spikes *(BECTS), and has since been changed to *benign rolandic epilepsy* (BRE) because it has been better defined. The subsequent proposal for classification in 2001 [**10,19**], suggests that the BRE and Panayiotopoulos syndrome are better classified as ”conditions with epileptic seizures that do not require a diagnosis of epilepsy.” This was a new concept proposed in the ILAE classification of 2001. One-third of children with BRE exhibit unique seizures, which do not fit with the classical definition of epilepsy (at least two seizures). The term benign also reflects the low frequency of seizures and epilepsy evolution towards spontaneous remission in adolescence [**10,11**]. Proposals for the classification of epileptic syndromes suggests that the term ”epilepsy of unknown cause” should replace the term ”cryptogenic epilepsy” and that ”there are reasons to be included in this group other forms of epilepsy previously considered idiopathic epilepsy, such as BRE and benign occipital epilepsies [**3**]. Many epileptic syndromes (including BRE) contain in their name “benign” or ”self-limited”, which refers to the syndromes’ good treatment response and self-limiting nature. In these forms of epilepsy, there may be associated cognitive and behavioral co-morbidities, psychiatric illness, migraine, or sudden death syndrome; and thus, the term ”benign” is not appropriate in these cases. Recent proposals for classification recommended the use of the term ”self-limited” instead of ”benign”. The characteristics of self-limited epileptic syndromes are as follows: a defined age of onset; seizures that are self-limited, meaning that the spontaneous remission has been reached; and the consequences of seizures, if they exist during the active phase of the disease, are not debilitating. However, it is not excluded an increased risk for cognitive or behavioral disorders of moderate intensity prior to the onset of seizures, during or beyond the active phase of the disease. The last classification proposal of epileptic syndromes includes BRE as a self-limited epileptic syndrome [**3**]. Taken together, although BRE has been identified for over half of a century, still remains as an ill-defined epileptic syndrome. The aim of this study was to evaluate if BRE children have cognitive deficits at the onset of their seizures, prior to their participation in any AED therapy.

## Methods

*Participants: *The BRE group consisted of 18 children who presented at least one rolandic-type seizure, did not undergo AED therapy and fulfilled the criteria for BRE according to the Commission of Classification and Terminology ILAE 1989 and 2001 [**8,9**]. The control group consisted of 18 age-matched healthy children. The inclusion criteria for the control group: the children were to exhibit no personal or familial history of epilepsy or other neurological disorders; the control children were to display a normal neurological examination, normal intellectual development for their age and a normal EEG recording. An informed consent was obtained from the family for the children’s participation in this study.

*Measures: *We recorded: 1) the seizures’ clinical features, 2) age of onset, 3) time of the day when the seizures appeared, and 4) seizure duration, which was subjectively evaluated from the history, obtained from the parents or other seizure witnesses. We considered that there is present an important emotional involvement, in particular, anxiety. A standard physical and neurological examination was performed on the patients and the control subjects. 

The EEG recordings were performed on the patients and controls, and consisted of 20 minutes of spontaneous awake recording with activation. The examination was performed with the child in the dorsal decumbent position, and in a silent and dimly lit environment. In the BRE group, we obtained spontaneous nocturnal sleep recordings. We used 19 surface electrodes placed according to a 10/20 International System. The centrotemporal spikes (CTS), were visually identified. We further analyzed the CTS according to the following characteristics: 1) its appearance as isolated or grouped in trains, 2) its frequency (on the basis of other studies, we classified the children into two groups, lower than 10/minute and higher than 10/minute), and 3) the presence of generalized epileptiform discharges, all of which were found in both awake and sleep EEG recordings. The children’s intellectual ability was measured by the Ravens Progressive Matrices. 

We used a computerized Cambridge Neuropsychological Test Automated Battery (CANTAB) for the neuropsychological assessment. The following tests were administered: 1) Induction: Motor Screening Test (MOT); 2) Visual memory: Delayed Matching to Sample (DMS), Paired Associates Learning (PAL), Pattern Recognition Memory (PRM), and Spatial Recognition Memory (SRM) tests; and 3) Executive functions: Spatial Span (SSP) and Spatial Working Memory (SWM) tests. A more detailed technical description of the tests may be found on the Cambridge Cognition’s website: http://www.cantab.com. The EEG recordings and neuropsychological assessment were not performed simultaneously, but in timed intervals of one to three days. The CANTAB assessment was performed at least three days after the last seizure and seizures’ rescue medication (Diazepam per rectal or intravenous application). 

*Statistical analysis: *All of the data were statistically analyzed using the mean ± standard deviation. We used a parametric test (t test - for independent samples) for normally distributed continuous variables and a Mann-Whitney U test for continuous non-parametric variables or small samples. A Pearson two-tailed correlation coefficient was applied to calculate agreements between the parameters. A p-value of < 0.05 was considered statistically significant. A Microsoft Excel software package and the Statistical Package for the Social Sciences (SPSS Inc Chicago, IL) were used for these calculations.

## Results

The BRE group consisted of 18 children (13 boys and 5 girls) with a mean age of 8.88 ± 2.37 years (between 6 – 14 years). All the children had one up to 3 seizures in their history and no AED therapy during the EEG and CANTAB assessments. Three of the patients received rescue medication (per rectal Diazepam) at the emergency department. The clinical and electroencephalographical characteristics are summarized in **Table 1**.

**Table 1:** Clinical and EEG characteristics of children from the BRE group

Clinical parameters		EEG parameters	
Consciousness lost during the seizure	12 (66.7%)	Predominant location of rolandic spikes	
Ictal/post-ictal aphasia	11 (61.1%)	Temporal	8 (44.4%)
Seizure duration	1.509 ± 2.298 min	Central	8 (44.4%)
Seizures that occur only during sleep	12 (66.7%)	Frontal	1 (5.6%)
Seizures that occur both during the sleep and awake state	6 (33.3%)	Parietal	1 (5.6%)
		Rolandic spikes lateralization	
		Left hemisphere	13 (72.22%)
		Right hemisphere	5 (27.77%)
		Rolandic spikes grouped in trains in the awake EEG	8 (44.4%)
		Rolandic spikes grouped in trains in the sleep EEG	11 (61.1%)
		Generalized SWC	2 (11.11%)
		Rolandic spike frequency in the awake EEG	11.222 ± 9.973 spikes/minute
		Rolandic spike frequency in the sleep EEG	27.055 ± 14.407 spikes/minute
Mean age: 8.88 ± 2.37 years (between 6 – 14 years), M:F = 2.6:1. Seizures onset mean age: 6.97 ± 1.86 years			

None of the children presented episodes of status epilepticus or Todd paralysis. All of the children in the BRE group were right-handed. All of the children exhibited an IQ that was higher than 85 (between 87 and 115) and no global intellectual deficits were shown in either of the groups. The CANTAB battery test results for BRE and the control group children are summarized in Table 2.

**Table 2:** CANTAB battery test results for the BRE and control group

Parameter	Patients (N = 18)		Control (N = 18)		t	p
	Mean	SD	Mean	SD		
Age	8.88	2.34	8.22	2.66		
IQ	93.77	7.59	95.44	8.12		
Induction						
MOT Mean Error	12.55	3.29	11.30	1.86	1.39	0.17
MOT Mean Latency	1120.70	166.10	1104.33	156.28	0.30	0.76
Visual memory						
DMS Percent Correct all Delays	68.18	14.72	71.11	16.95	-0.55	0.58
DMS Percent Correct Simultaneous	90.00	17.14	97.77	6.46	-1.80	0.08
DMS Probe Error Given Error	0.17	0.18	0.25	0.17	-1.44	0.15
PAL Total errors adjusted	14.55	16.37	7.88	5.06	1.65	0.10
PAL Total errors, 6 shapes adjusted	3.50	5.22	2.72	3.15	0.54	0.59
PAL First Trial Memory Score	19.27	3.70	20.22	2.81	-0.86	0.39
PAL Mean errors to success	1.87	2.18	0.98	0.63	1.65	0.10
PAL Mean trial to success	1.67	0.57	1.41	0.22	1.75	0.08
PAL Stages completed on first trial	5.72	0.89	5.88	0.83	-0.57	0.56
PRM Mean correct latency	2922.74	513.02	2644.43	711.67	1.34	0.18
PRM Percent correct	81.24	16.80	91.89	4.84	-2.58	0.01
SRM Mean correct latency	2902.04	1118.91	2949.29	621.92	-0.15	0.87
SRM Percent correct	71.88	11.09	79.72	4.99	-2.73	0.01
Executive functions						
SSP Span length	4.55	1.09	4.56	1.42	0.01	0.99
SSP Total errors	11.38	5.11	9.94	2.71	1.05	0.29
SWM Between errors	43.55	20.94	38.16	21.12	0.76	0.44
SWM Double errors	0.88	1.02	1.38	2.63	0.75	0.45
SWM Strategy	34.27	4.90	34.33	5.20	0.03	0.97

The BRE group exhibited normal performances for induction and executive functions, compared with control children. There were present significant differences for the visual memory subtests. The PRM percent correct (t = -2.58, p = 0.01) and SRM percent correct (t=-2.73, p=0.01) indicate that the BRE children had difficulties in determining the accuracy of the pattern and spatial recognition memory. There were no significant correlations between the CANTAB test results and clinical parameters (ictal/post-ictal aphasia, consciousness during the seizure, the seizures’ occurrence on sleep and/or the awake-state, the seizure’s duration). There were significant correlations between the onset age for the seizures and visual memory (PRM mean correct latency, r = -0.59, p < 0.05 and close to significance for the SRM mean correct latency, r = -0.32). We have identified statistically significant correlations between the CANTAB test results and specific EEG parameters. **Induction: **The MOT mean latency results are directly related to the CTS grouped in the trains of the awake state (t = 2.15, p < 0.05; U = 20, p = 0.07) and the frequency of the CTS during sleep (r = -0.51, p < 0.05). **Visual memory:** The CTS grouped in the trains during sleep influences the performance of visual short-term recognition memory (PRM percent correct: t = 2.18, p = 0.04; U = 19.50, p = 0.07) and the frequency of the CTS on the awake state was correlated with PAL: first trial memory score (r = 0.48, p < 0.05). The appearance of the CTS on the right hemisphere influenced PAL stages completed on the first trial (t = 2.20, p < 0.05; U = 13, p < 0.05). **Executive functions: **The CTS frequency alters the SSP span length, a subtest for executive functions (U = 18.5, p < 0.05). In the BRE group, the CTS dominantly appeared in the temporal (8 children) and central (8 children) regions, less in frontal and parietal region (1 child in each). We compared the CANTAB test results for temporal and central CTS dominance and found no differences between the two groups. 

Limitations of the study: we performed limited subtests from the CANTAB battery of exams. We were not able to investigate attention, reaction time, verbal memory, and decision-making or response control. Our future directions include the investigation of these parameters in the relationship with epilepsy at the onset of the seizures. In addition, the sample size was reduced in this study (although this is typical for studies in child epilepsy). 

## Discussions

Although BRE children have normal IQ ranges, statistically it was reported to be lower than in controls; 72.7% of them had difficulties in at least one test administered [**20**]. We found no major neuropsychological deficits, except for impairments in visual memory (pattern and spatial recognition memory). Similar results have been found by previous studies in BRE children; other studies (using other neuropsychological tests) have found lower performances in visual-motor coordination, image naming and disorders of visual perception abilities [**16,5,2,1,20**]. These visual disorders were reported to be dependent on the localization of the CTS [**7,13,15,16,21**]. Additionally, a higher CTS frequency was associated with more severe transient cognitive disorders induced by subclinical epileptiform activity [**20**]. Thus, the lower performances on motor tasks are due to deficits in the visual-motor adaptation [**4,16,19,12,6**]. These neuropsychological deficits could be due to the epileptic syndrome, or they could be the effect of cognitive plasticity in response to focal functional impairment, [**21**]. It was previously thought that neural networks in these children’s brains were not sufficiently developed for higher cognitive functions [**16,18**]. In support of this hypothesis is the correlation we found between visual memory tests (PRM – mean correct latency and potential SRM – mean correct latency) and the age of seizure onset. We found correlations between the different neuropsychological functional results and subclinical epileptiform discharges. The MOT mean latency was altered by the frequency of the CTS in sleep and the presence of the CTS grouped in trains in the awake stages. In Landau-Kleffner syndrome, a complicated form of BRE, there are reports of associated motor impairment in the form of dyspraxia, dystonia, ataxia, or unilateral deficits [**16,17**]. In addition, interictal paroxysmal activity may interfere with different cognitive processes, as demonstrated by neurophysiological, neuropsychological, and biochemical studies; the frequency of the CTS is proved to play a role in these cognitive deficits [**1**]. It was also identified that the CTS grouped in large clusters/trains, represents an unfavorable evolutionary marker of BRE, with a higher risk for the development of neuropsychological deficits [**22**]. Studies using simultaneous acquisitions of EEG and fMRI in children with continuous spikes and waves during slow-sleep, showed that patients demonstrated highly significant, spike-related activations and deactivations of blood oxygenation-level-dependent changes. These activations involved the bilateral perisylvian region and the cingulate gyrus in all of the subjects, as well as the bilateral frontal cortex, bilateral parietal cortex and thalamus in some of the subjects, demonstrate the activation of similar networks in these children, in particular, the perisylvian region, insula and cingulate gyrus [**18**]. 

We also identified correlations that were closely significant between the subtests for working memory (SWM strategy, SWM between errors and SWM double errors) and the frequency of CTS in the awake or sleep state. The SWM is a sensitive measure for executive dysfunction of the frontal lobe, which is not generally involved in BRE. Functional MRI and PET studies in normal subjects, that evaluated non-spatial or spatial working memory, have provided strong pieces of evidence that the prefrontal cortex is involved in working memory. Moreover, additional studies have reported the involvement of the occipital lobe (to create an internal image of the target), parietal cortex (to calculate the target’s coordinates), and the prefrontal cortex (to store the target and to retain the image of the memory during the retention interval) in working memory. The activation of other brain regions has often been observed during the performance of working memory tasks (posterior parietal cortex, Broca’s area), suggesting that working memory function is sub served by multiple brain regions. Visual memory is also a complex function supported by fronto-parietal and visual cortical neurons, interacting via newly formed functional connections among these regions [**14**]. 

Globally, the results of our study are consistent with the results of previous studies. We found that BRE children commonly display normal ranges of global cognitive development. However, neuropsychological assessments reveal more subtle deficits. An early seizure onset has a negative impact on visual memory development (and potential effects on other cognitive functions as well). In particular, the correlations we found between the test results and EEG parameters provide evidence that BRE is not strictly characterized by focal dysfunction. There is a functional involvement of larger areas and networks of the brain, which can be subsequently associated with various neuropsychological deficits, including those that are characterized as “mild”, which are generally not reported by parents or teachers.
